# Exploring the multiphysics of the brain during development, aging, and in neurological diseases

**DOI:** 10.1016/j.brain.2023.100068

**Published:** 2023-04-24

**Authors:** Johannes Weickenmeier

**Affiliations:** Department of Mechanical Engineering, Stevens Institute of Technology, Hoboken, NJ 07030, United States of America

**Keywords:** Multiphysics brainmechanics, Brain aging, Neurodegenerative disease, Mechanical testing, Longitudinal medical image registration

## Abstract

The human brain remains an endless source of wonder and represents an intruiging scientific frontier. Multiphysics approaches naturally lend themselves to combine our extensive knowledge about the neurobiology of aging and diseases with mechanics to better capture the multiscale behavior of the brain. Our group uses experimental methods, medical image analysis, and constitutive modeling to develop better disease models with the long-term goal to improve diagnosis, treatment, and ultimately enable prevention of many prevalent age- and disease-related brain changes. In the present perspective, we outline on-going work related to neurodevelopment, aging, and neurodegenerative disease.

## 1. Introduction

The human brain undergoes a myriad of changes during its lifetime. From a mechanics perspective alone, it is mesmerizing how the brain develops during early life, transforms into this highly functional, albeit still very enigmatic, organ that makes us unique, and is subjected to injury, disease, and ultimately age-related degeneration like every other part of the body. Despite extensive efforts to mechanically characterize brain tissue for more than two decades, literature continues to provide a wide range of experimental results [1,2]. Additionally, the relationship between microstructure, state of health, and mechanical behavior remains elusive. On the modeling side, the computational biomechanics community has had extensive interest in modeling traumatic brain injury and developed theories and simulation approaches to investigate brain impact and blast injury [3,4]. Only in the past decade, have other applications emerged that range from brain tumor growth, to neurodegeneration, stroke, surgical guidance, and, the most intensely studied, brain folding during early development [5–12].

Our group’s motivation to pursue multiphysics modeling of the brain is simple: while the biology of brain aging and many neurological diseases is very well established, its coupling to the brain’s mechanical response in the form of cerebral atrophy and tissue degeneration/damage remains understudied. We are focusing on (i) multiphysics methods to integrate either neurobiology or medical image-derived data into our computational modeling approaches, (ii) developing new models that capture morphological changes during aging, (iii) exploring the role of mechanics as a major contributor to age-related neurodegeneration, and (iv) further investigating the mechanical behavior of brain tissue to resolve existing contradictions reported in literature. In the following, we briefly outline our recent work.

## 2. Computational brainmechanics

### 2.1. Inferring brain growth fields during brain development from longitudinal image data

Early human brain development is characterized by neuronal cell proliferation, migration, and aggregation in the periphery of the outer cortical layers. The gradual densification of arriving neuronal cells and the subsequent formation of a hyperdense inter-neuronal dendritic network causes mechanical folding. The repeatability of the resulting folding patterns across every brain is attributed to migration pathways that are genetically encoded in the emerging brain. Current modeling approaches use multifield finite element formulations to integrate this knowledge into their constitutive models of brain growth. The computational mechanics community uses the multiplicative split of the deformation gradient to prescribe volume change, i.e., dilatation, and obtain the compatible purely elastic deformation that is associated with stress [8].

**Fig. 1.**
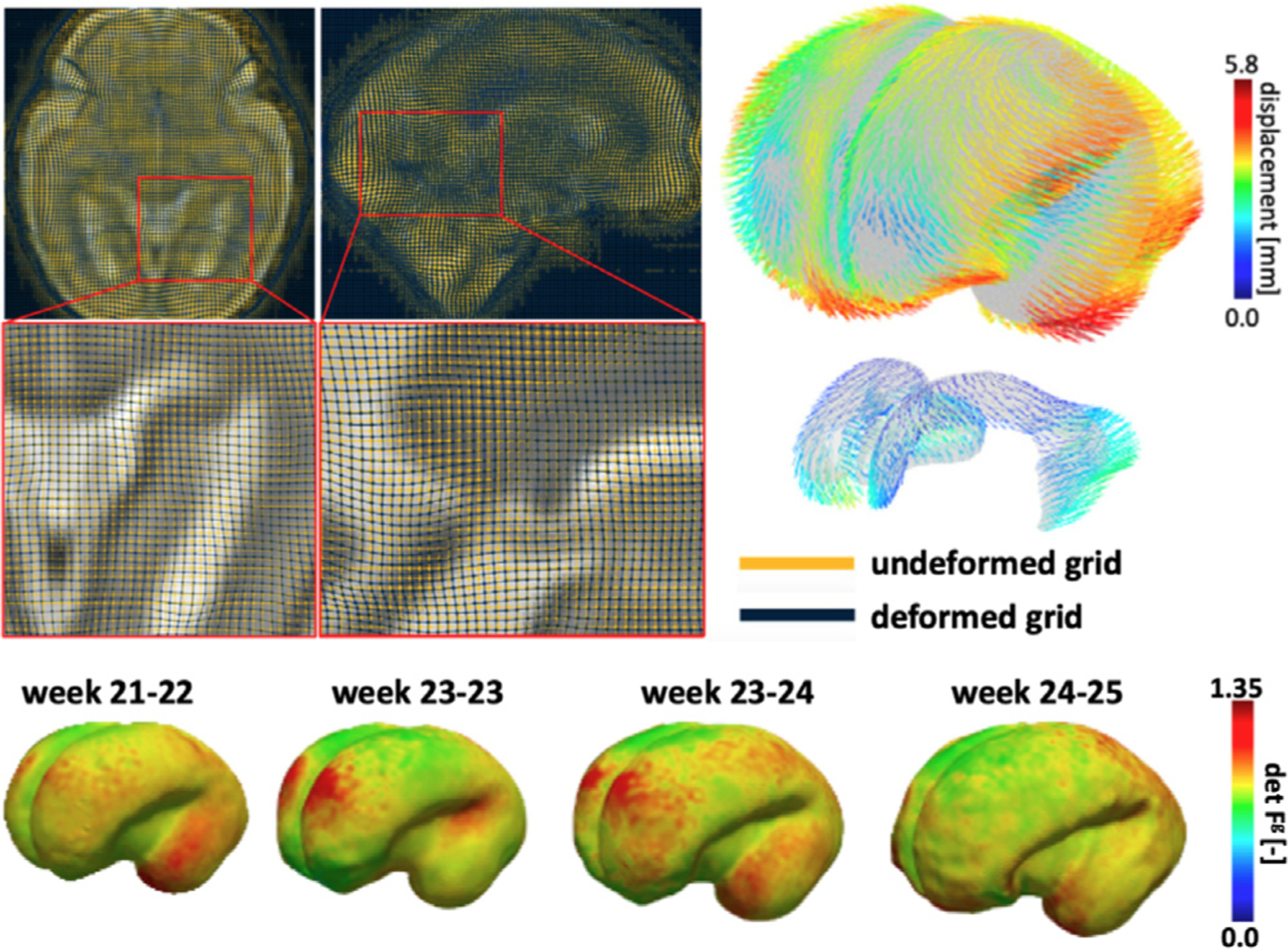
We infer the whole brain growth field based on registration results from longitudinal image data and visualize the results on the ventricular and cortical surfaces [13].

A limitation of existing work is the lack of validation of simulation results against experimental or clinical data. We recently sought to address this issue by using a fetal brain atlas to determine brain deformations between two consecutive weeks on the one hand, and an inverse finite element modeling approach to infer the volumetric brain growth field on the other [13]. Instead of prescribing the volumetric growth field in the entire brain, our approach delivers the data-derived growth field that satisfies the theory of morphoelasticity, see Fig. 1. For the first four weeks of the atlas, our study showed that brain growth is highly heterogeneous with respect to both space and time. During early brain growth, we notice consistent volumetric growth across the whole brain. During later weeks, brain growth transitions to mostly tangential expansion of the cortical layer [13]. These findings are important confirmation of the existing theory of brain growth and provide new insight into the temporal growth patterns of the developing brain. More specifically, the proposed framework can be applied to other examples for which longitudinal image data exists.

### 2.2. Predicting age-related brain shape changes and corresponding neuropathology

**Fig. 2.**
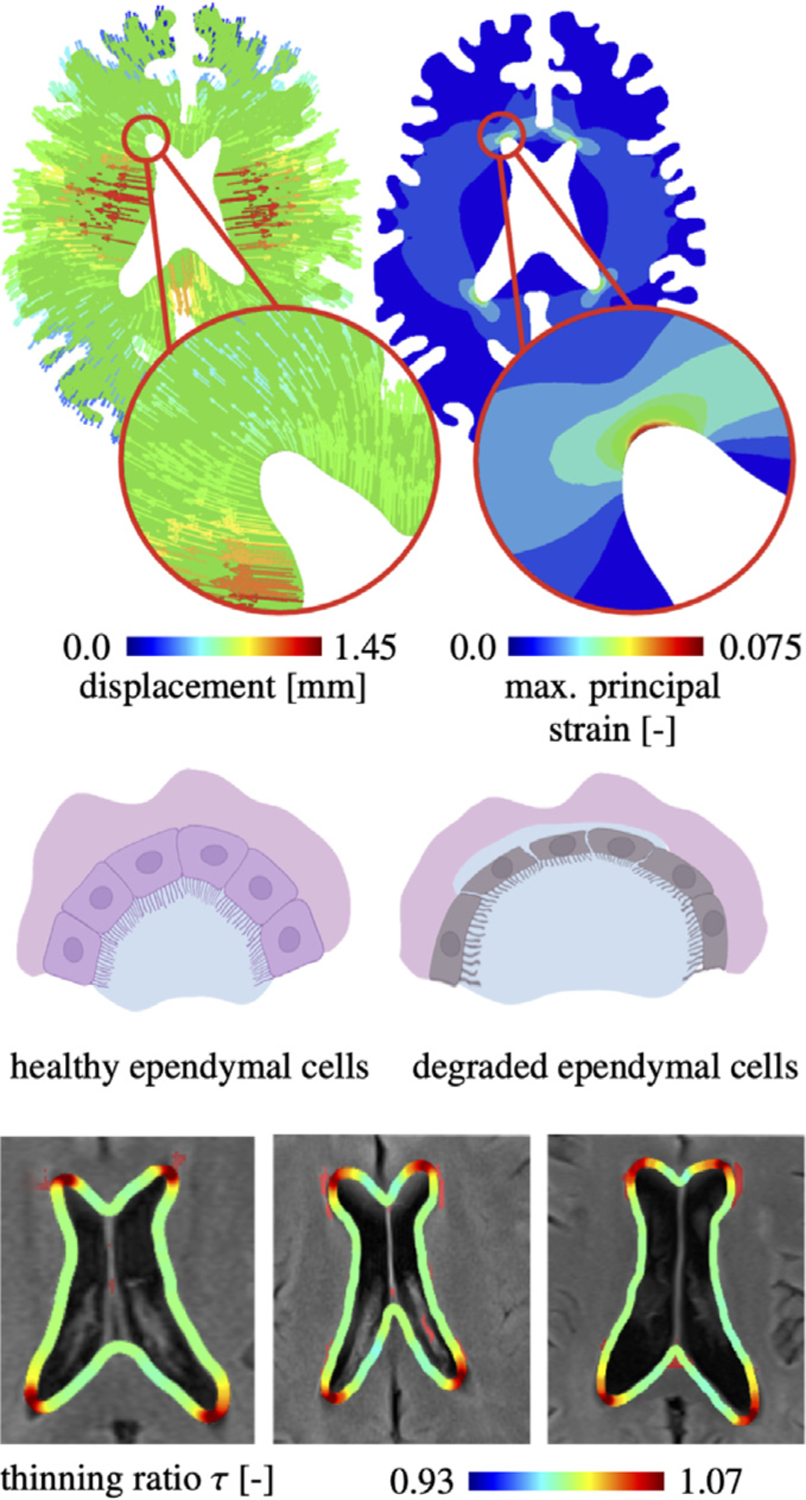
Age-related ventricular expansion causes ependymal cells in the ventricle’s horns to experience loads which lead to their functional decline and CSF-leakage into nearby white matter [21,22].

Like all organs, the brain is affected by age and functional decline [14,15]. The underlying brain changes are mostly associated with the gradual degeneration of cellular microstructure due to the loss of dendritic connections in gray matter [16] and neuroinflammation-driven demyelination, axon death, astrogliosis and microgliosis in white matter [17]. From our modeling work on Alzheimer’s disease, see Section 2.3, we have come to realize that cerebral atrophy causes extensive mechanical loading in many brain regions known to suffer from age-related damage [18]. More specifically, some of the prominent features of aging [19], such as cortical thinning, ventricular enlargement, and sulcal widening, create significant levels of strain across the aging brain. Accompanying deterioration of tissue integrity leads to significant functional decline [20]. For example, we have observed that ventricular enlargement creates particular strains on periventricular white matter tissues which spatially correlate with lesions known as periventricular white matter hyperintensities [21,22], see Fig. 2. These lesions appear as bright spots on fluid attenuated inversion recovery (FLAIR) imaging in subjects of advanced age [23]. Interestingly, they consistently form in the horns and along the edges of the lateral ventricle’s main body. More importantly, the ventricular wall is made up of cuboidal ciliated ependymal cells that form a very tight functional barrier between brain tissue and cerebrospinal fluid that is produced in the choroid plexus of the ventricles and drains into the subarachnoid space through the 3rd and 4th ventricles due to cardiac- and respiratory-driven forces [24]. The cuboidal cells are connected via tight cadherin and other gap junctions that actively regulate the fluid and nutrient transport across this crucial barrier [25]. We demonstrated that cerebral atrophy causes these cells to be stretched thin [21,22] and, therefore, hypothesize that age-related ventricular enlargement eventually causes fluid to leak into the periventricular space and trigger white matter lesion formation. We are currently coupling our atrophy model to a periventricular white matter hyperintensity damage field to predict the progressive growth of periventricular white matter hyperintensity volumes. This work challenges existing notions that age-related white matter lesions are purely driven by neurobiology when, in fact, mechanics represents another important source for neurodegeneration [19].

### 2.3. Assessing the impact of Alzheimer’s disease on brain shape

Alzheimer’s disease and related dementias represent a major health threat and significant societal burden due to a progressively aging population. The disease’s extensive pre-symptomatic progression period is a major obstacle to early intervention when treatments are most effective in delaying the onset of symptoms. There is a dire need for diagnostic and health monitoring tools that allow to detect abnormal brain changes as early as possible in order to affect disease outcome. We have been developing anatomically accurate finite element brain models to predict brain changes in healthy and accelerated aging to detect characteristic morphological features associated with early disease phases [18,26]. More importantly, we are developing multiphysics aging models that differentiate between healthy aging and disease-related acceleration of neurodegeneration. More specifically, we use a two field finite element formulation to capture brain deformations, driven by cerebral atrophy-based tissue shrinking, and toxic protein progression associated with the progression of neurofibrillary tangles from misfolded tau [18,26]. The two fields are coupled in that the presence of tangles increases the atrophy rate and locally accelerates tissue shrinking. Atrophy is governed by the theory of morphoelasticity and tangle progression is governed by the well-known Fisher–Kolmogorov equation, i.e., a reaction–diffusion problem developed to simulate population growth [18,26].

**Fig. 3.**
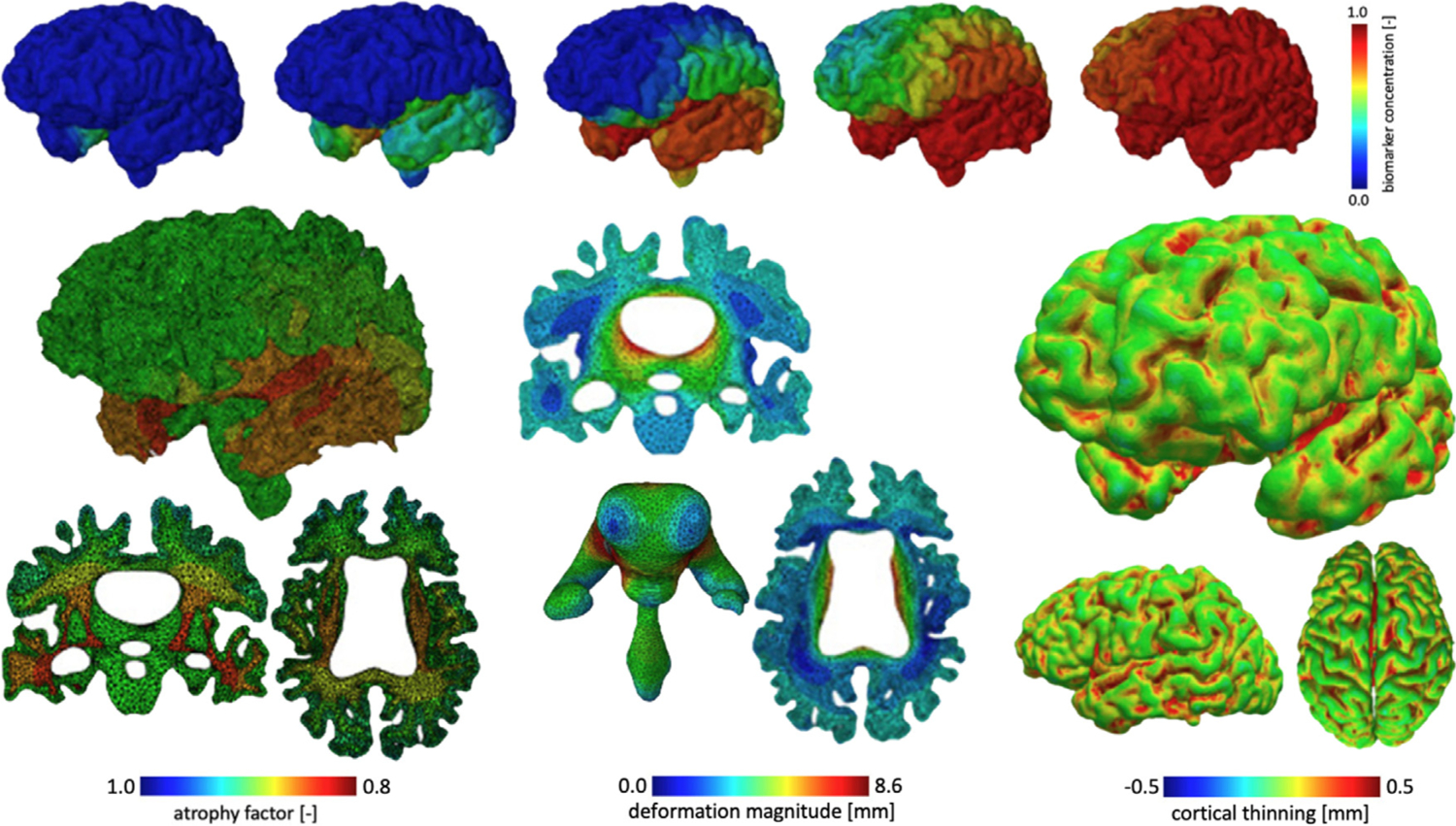
We simulate biomarker progression and cerebral atrophy during aging. Our model reproduces hallmark features such as sulcal widening, ventricular enlargement, and cortical thinning [18].

Using our whole-brain modeling approach, we have been able to reproduce a number of characteristic morphological and protein-related features linked to Alzheimer’s disease and related dementias, see Fig. 3. On the one hand, our models capture the clinically-observe toxic protein spatiotemporal progression patterns of *β*-amyloid hyperphosphorylated tau in Alzheimer’s disease, *α*-synuclein in Parkinson’s disease, and TDP43 in amyotrophic lateral sclerosis [27,28]. On the other hand, the atrophy-related part of our model induces cortical thinning, sulcal widening, gray and white matter volume loss, and ventricular enlargement to an extent commonly observed in population-based imaging studies [18]. Interestingly, our model allows to identify the inflection point when Alzheimer’s disease-related brain changes deviate from healthy aging patterns.

## 3. Experimental characterization of the brain

We are predominantly interested in brain stiffness changes associated with brain aging and disease. Previous work has illustrated substantial brain stiffening during early development and that the brain softens during aging and with the progression with neurodegenerative disease [29–31]. It has also been shown that metabolic brain activity, i.e., noticeable changes in brain homeostasis, influences brain stiffness [32,33]. In general, it is known that gray matter in rodents is significantly stiffer than white matter [34], whereas in larger mammals and humans white matter is roughly two times stiffer than gray matter [35]. Extensive work has gone into micromechanical characterization of distinct brain regions such as the hippocampus [36], corpus callosum [34], spinal cord [37,38], meninges [39].

**Fig. 4.**
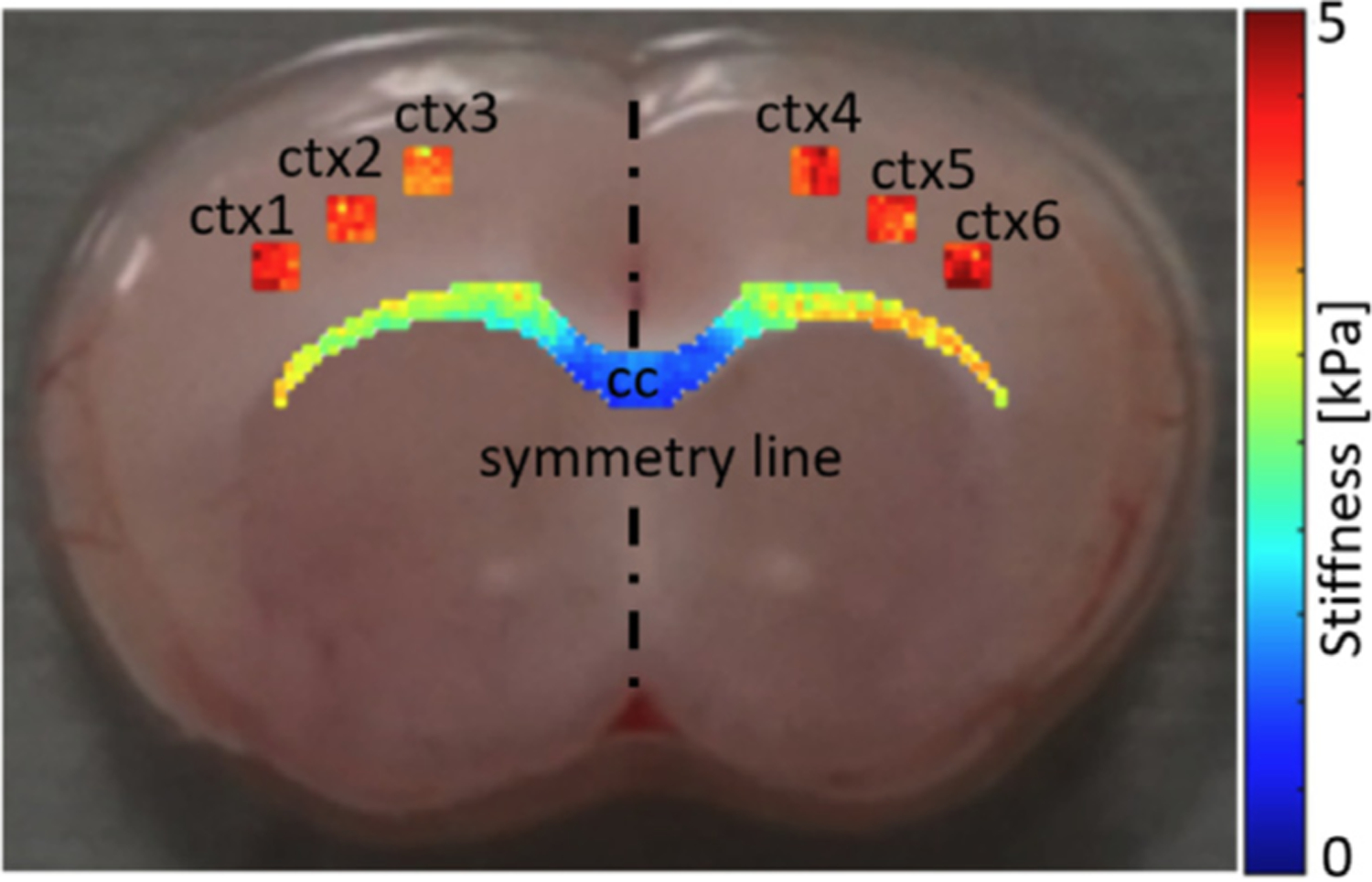
Tissue stiffness in mouse brain corpus callosum and cortex from microindentation measurements.

Our work has shown that myelin density is a sensitive marker for brain tissue stiffness [40,41]. Although it does not predict absolute tissue stiffness, myelin content is highly proportional to local tissue stiffness. Myelin mostly appears in white matter where it wraps around axons to increase signaling speed and provides mechanical integrity to the axon. Axonal extensions in the cortex have considerably less myelin which, therefore, plays a significantly lower role in comparison to neuron cell density and number of dendritic connections [42]. Besides extensive stiffness variations across the corpus callosum in mice brains, see Fig. 4, we observed significant transient tissue stiffness changes in response to fluctuations in myelin density (work currently under review). This is particularly relevant to demyelinating diseases which are often associated with recurring flares of neuroinflammation that result in temporary demyelination such as in multiple sclerosis. Developing interventions that facilitate neuronal and myelin repair depend on a reliable assessment of the mechanical microenvironment conducive to cell proliferation and differentiation into myelin-depositing oligodendrocytes in lesion sites.

## 4. Current challenges and opportunities in brain multiphysics

Multiphysics modeling offers an intriguing approach to studying the complex nature of the brain. Current opportunities are understanding the relationship between mechanical properties and microanatomy, verification and validation of novel computational models, and developing accurate neurological disease models.

### 4.1. Resolving the relationship between tissue composition, microanatomy, and mechanical behavior

Resolving the structure–function relationship of neuronal tissues is an important step towards a better understanding of brain health. Ageand disease-related neurodegeneration and brain injury have various effects on brain microanatomy and inherently decrease functional performance. In order to develop more effective treatment methods for neuronal repair following damage or disease, we need to fully establish the mechanobiological properties of brain tissues using experimental and computational methods. Combined analysis of biological and mechanical properties is paramount to also capture the properties as they evolve with maturation, aging, and disease.

### 4.2. Validating computational models against experimental and clinical data

Computational models play a critical role in predicting brain behavior under various states of health. Their rigorous validation against clinical or patient data, however, continues to be mostly absent from literature. Medical imaging is one of the most frequently used clinical tools to monitor brain health. For one, its integration into future simulation frameworks will enable validation of models and simulations. For the other, integration of imaging data will allow to calibrate or infer the constitutive models of the neuropathological mechanisms that manifest in brain changes.

### 4.3. Developing multiphyics-based neurological disease models

Multiscale and multiphysics modeling provides an intriguing avenue to provide new insights into many neurological diseases that present with complex spatial and temporal pathological progression patterns. By the time that many diseases develop symptoms that allow for definitive diagnosis, neuropathology has often already progressed to an advanced stage. Earlier diagnosis remains nearly impossible, however, because it would require frequent monitoring of an otherwise healthy population from early age. Computational disease models can help identify early brain changes, inform potential intervention strategies, and become an inexpensive monitoring tool in the future.

## 5. Summary

Our work focuses on developing novel experimentally-informed and data-driven computational models to simulate brain aging and age-related neurological diseases. Our goal is to better understand the relationship between neuropathology and organ-level brain changes that are visible in *in vivo* medical images such as MRI. We use multiphysics modeling approaches to couple well understood biological disease mechanisms to mechanical tissue changes ranging from atrophy to damage.

## Data Availability

All data presented in this perspective are available upon request.
